# FocaL mass drug administration for *Plasmodium vivax *malaria elimination (FLAME): study protocol for an open-label cluster randomized controlled trial in Peru

**DOI:** 10.1186/s13063-025-09112-1

**Published:** 2025-10-14

**Authors:** Sydney R. Fine, Veronica Soto Calle, Astrid Altamirano Quiroz, Hugo Rodriquez Ferruci, Paulo Manrique, Xue Wu, Gabriel Carrasco Escobar, Jade Benjamin-Chung, Adam Bennett, Sarah Auburn, Ric N. Price, Bryan Greenhouse, J. Kevin Baird, Gonzalo J. Domingo, Michelle E. Roh, Angel Rosas, Alejandro Llanos-Cuentas, Michelle S. Hsiang

**Affiliations:** 1 Malaria Elimination Initiative, Institute for Global Health Sciences, University of California San Francisco (UCSF), San Francisco, CA USA; 2 Unidad de Leishmaniasis y Malaria, Instituto de Medicina Tropical Alexander von Humboldt, Universidad Peruana Cayetano Heredia, Lima, Perú; 3 Department of Immunology and Infectious Diseases, Harvard T.H. Chan School of Public Health, Boston, MA USA; 4 Department of Epidemiology and Population Health, Stanford University, Stanford, CA USA; 5 Department of Epidemiology and Biostatistics, UCSF, San Francisco, CA USA; 6 PATH, Seattle, WA USA; 7 Menzies School of Health Research and Charles Darwin University, Darwin, 0811 NT Australia; 8 Centre for Tropical Medicine and Global Health, Nuffield Department of Medicine, University of Oxford, Oxford, OX3 7LJ UK; 9 Mahidol-Oxford Tropical Medicine Research Unit, Faculty of Tropical Medicine, Mahidol University, Bangkok, 10400 Thailand; 10 EPPIcenter Program, Division of HIV, ID and Global Medicine, University of California, San Francisco, CA USA; 11 Oxford University Clinical Research Unit, Faculty of Medicine Universitas Indonesia, Jakarta, Indonesia; 12 The Centre for Tropical Medicine and Global Health, Nuffield Department of Medicine, University of Oxford, Oxford, United Kingdom; 13 Department of Epidemiology, Division of Infectious Diseases and Global Health, Department of Pediatrics, Division of Pediatric Infectious Diseases, UCSF, San Francisco, CA USA

**Keywords:** Chemoprevention, Tafenoquine, Primaquine, Glucose 6 phosphate dehydrogenase deficiency, Cluster randomized control trial, Peru, Malaria, *Plasmodium vivax*

## Abstract

**Background:**

Outside of sub-Saharan Africa, *Plasmodium vivax* has become the dominant species of malaria. Focal mass drug administration (fMDA) is a potential strategy to support elimination efforts, but controlled studies are lacking.

**Methods:**

The FocaL mass drug Administration for *Plasmodium vivax* Malaria Elimination (FLAME) study is a 3-year cluster randomized controlled trial to determine the impact and safety of fMDA to reduce *P. vivax* transmission. The study will be conducted in Loreto, Peru, where standard interventions have reduced *P. vivax* cases, but transmission persists due to a high proportion of subclinical infections. Thirty low transmission communities (API < 250 cases/1000 population) will be randomized 1:1 to fMDA versus control using a restricted randomization. All communities will receive Peruvian national standard malaria control measures. In the intervention arm, high-risk individuals (living within 200 m of a *P. vivax* case reported in the prior 2 years) without contraindication to study medications, including G6PD deficiency, will receive three cycles of fMDA over a 2-year period. Each cycle will include two rounds of directly observed therapy delivered 2 months apart. The fMDA regimen will include 25 mg/kg chloroquine (CQ) plus a single 300 mg dose of tafenoquine (TQ) for individuals age ≥ 16 years, and 25 mg/kg of CQ plus 7 days of 0.5 mg/kg/day of primaquine (PQ) if younger. The primary outcome is the cumulative incidence of symptomatic *P. vivax* malaria. The sample size provides 80% power to detect at least a 68% relative reduction in cumulative *P. vivax* incidence, based on alpha of 0.05 and a coefficient of variation (*k*) of 0.87. Secondary outcomes include safety, cost-effectiveness, and infection prevalence and seroprevalence which will be assessed in annual cross-sectional surveys. Safety will be assessed in passive and active pharmacovigilance, including post-treatment screening for G6PD-associated hemolysis by assessing for anemia and hematuria in a sample.

**Discussion:**

The trial will generate evidence regarding fMDA for *P. vivax* and inform malaria elimination efforts in Peru and similarly endemic settings. Findings will be disseminated in peer-reviewed publications and through stakeholder meetings in Peruvian and international research forums.

**Trial registration:**

Clinicaltrials.gov NCT05690841. This trial was registered on 09 January 2023. Peruvian Clinical Trial Registry (REPEC) 020–23. This trial was registered on 21 February 2024.

**Supplementary Information:**

The online version contains supplementary material available at 10.1186/s13063-025-09112-1.

## Background

Current malaria control interventions disproportionally reduce malaria due to *Plasmodium falciparum*, leading to an increase in the proportion of malaria due to *Plasmodium vivax*. Outside of sub-Saharan Africa, *P. vivax* has become the predominant cause of malaria [1–3]. *P. vivax* presents unique challenges for malaria elimination [4]. As with *P. falciparum* malaria, subclinical infections do not come to the attention of the standard health care system. However, unlike *P. falciparum* which invades all red blood cells, *P. vivax* tends to invade reticulocytes, resulting in relatively lower density infections that can be missed by standard diagnostics. Untreated, *P. vivax* persists in its blood stage and remains latent in the liver as a hypnozoite, a stage that reactivates weeks to months later causing symptomatic attacks called relapses [5, 6].

Primaquine is the only widely available drug that kills *P. vivax* hypnozoites, but its widespread use is undermined by safety, adherence, and efficacy concerns [7]. Primaquine can trigger severe hemolysis in individuals with inherited glucose-6-phosphate-dehydrogenase (G6PD) deficiency [8]. Adherence to the prolonged 7−14 day treatment course is often poor [9], and even complete adherence to standard regimens can lead to recurrent episodes of malaria in approximately 20% of cases [10]. These challenges in case management limit the effectiveness of active case detection approaches [11] and contribute to a reservoir of infections that perpetuate ongoing community transmission.

Mass drug administration (MDA) is a strategy that clears infections that would not otherwise be detected and treated. This approach is focused on the provision of antimalarial treatment to a defined population, irrespective of the presence of symptoms or infection, often repeated at intervals. In 2015, the World Health Organization (WHO) recommended the use of MDA to reduce *P. falciparum* transmission in very low to low transmission settings, defined as areas where prevalence is less than 10% or incidence is less than 250 cases per 1000 population per year, where coverage of standard interventions is high, and risk of importation is low [1, 12]. A recent review of MDA for *P. falciparum* identified eight cluster randomized controlled trials (CRCT) showing short-term impact [13]. Sustained impact is more likely when baseline transmission intensity is lower, but in these settings where most of the population is not infected and a high proportion of infected individuals have minimal or no symptoms, the risks of adverse events following MDA may outweigh its benefits. Decreased perception of risk can also lead to poor acceptability and adherence [14, 15]. Limiting MDA to the highest risk groups can minimize these drawbacks, while still being effective. Reactive drug administration, or MDA directed at foci of household members and neighbors of index cases was studied in four recent CRCTs that demonstrated it to be effective for reducing community-level transmission of *P. falciparum* [13, 16]. The focal application of MDA to specific higher-risk subpopulations also facilitates higher coverage of MDA, efficient and cost-effective use of limited resources, and safety monitoring [17, 18]. However, the logistical challenges associated with delivery of MDA through this reactive, or “on-call,” approach may preclude its use in many settings.

For *P. vivax*, there are no published CRCTs of MDA using radical cure administered either at a village-level or focally. Anecdotal evidence suggests that large-scale mass drug administration to entire communities contributed to *P. vivax* elimination in temperate settings including Azerbaijan, Tajikistan, Afghanistan, North Korea, and China [19, 20]. However, more focal approaches may be an effective strategy that can address the logistical and safety challenges associated with MDA using radical cure—namely the long treatment courses and the potential for G6PD deficiency-associated hemolysis [18]. Most *P. vivax* cases are relapses [21, 22], and relapsing infections can persist for many months and even years. Hence, an obvious strategy would be target individuals harboring hypnozoites [21, 23]. In the absence of a convenient diagnostic test to detect hypnozoites [21, 24], an approach used in Central China was to conduct MDA annually in households of and near index cases reported from the prior 1–2 years. Specifically, focal MDA (fMDA) was conducted annually preceding the high transmission season, which was eventually followed by sustained interruption of *P. vivax* [20]. Operational advantages of this approach are that it self-tailors to changes in transmission levels and the program has time to prepare for delivery that is proactive rather than reactive. Despite lack of controlled data, the WHO made a conditional recommendation for the use of MDA to reduce *P. vivax* transmission given the urgent need for new approaches to eliminate malaria. At the same time, the WHO called for further research on its impact, operational factors, safety, and feasibility, particularly in tropical or subtropical settings [1, 25].

To generate evidence to inform global *P. vivax* elimination efforts, building on the approach used in Central China, we propose a trial to evaluate the impact and safety of fMDA directed at households in proximity of *P. vivax* index cases from the prior 2 years. The Loreto region in the Peruvian Amazon was selected as the study site due to its low endemicity of malaria, predominance of *P. vivax*, preliminary data demonstrating cases are primarily subclinical and low density infections, and strong infrastructure to facilitate delivery of fMDA and measure its impact [26]. Importantly, Peru recently approved the use of tafenoquine (TQ), a single dose 8-aminoquinoline which has been shown to be non-inferior to primaquine (PQ) for radical cure, and a new point-of-care test for G6PD deficiency for symptomatic case management. These tools hold promise for facilitating safe and effective delivery of radical cure in communities as part of MDA for *P. vivax* transmission reduction and elimination [27].

### Aims and objectives

The overall study objective is to evaluate the impact and safety of fMDA for *P. vivax* transmission reduction compared to no fMDA. The primary aim is to determine the effect of fMDA on the outcome of cumulative incidence of symptomatic, microscopy-confirmed *P. vivax* cases over the study period. *P. vivax* incidence will be determined based on the number of laboratory-confirmed, locally acquired cases reported from health facilities serving the catchment area of study villages. Secondary outcomes include incidence of *P. falciparum* or *P. vivax* and *P. falciparum* and infection prevalence and seroprevalence of *P. vivax* and/or *P. falciparum* measured in cross-sectional surveys (Table S1). Secondary aims are to assess the safety, tolerability, and acceptability of fMDA, as well as its cost-effectiveness compared to current standard-of-care interventions. Safety and tolerability outcomes will be measured as incidence of adverse events detected through active and passive pharmacovigilance and vomiting after administration. Acceptability will be measured as refusal rates and reported willingness to participate in future fMDA campaigns. Cost-effectiveness will be measured as cost per case averted, or per disability adjusted life year (DALY) averted. We hypothesize that fMDA will result in a greatly reduced latent hypnozoite reservoir and that will translate to reduced incidence, infection prevalence, seroprevalence, and cost-effectiveness compared to standard interventions and that it will be safe, well-tolerated, and acceptable to the community.

## Methods and design

**Fig. 1:**
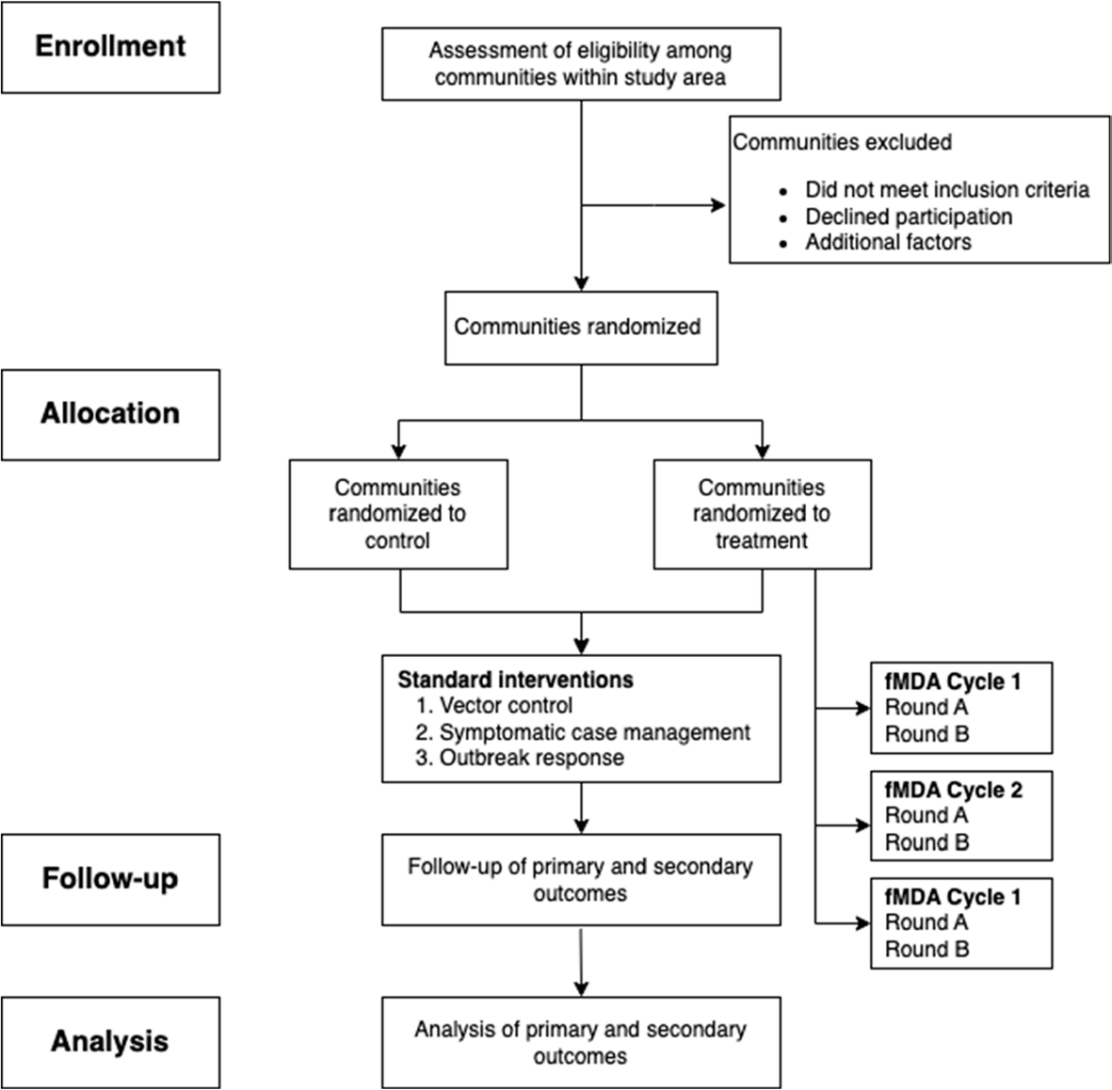
Flowchart demonstrating the involvement of FLAME communities throughout the trial per SPIRIT guidelines. Vector control includes the distribution of insecticide-treated nets (ITNs) and the use of indoor residual spraying (IRS). Symptomatic case management involves testing and treating confirmed malaria cases by microscopy or PCR testing at local health facilities or in the community by a community health worker. Outbreak response may include an active search strategy through screening and treatment of confirmed cases when malaria case incidence is increased significantly compared to the regular trend of cases for 2 consecutive weeks

The SPIRIT (Standard Protocol Items: Recommendations for Interventional Trials) recommendations were referenced in developing this protocol [28]. Figure 1 demonstrates the overall study communities’ involvement in the FLAME trial per SPIRIT guidelines.

### Study design

**Fig. 2:**
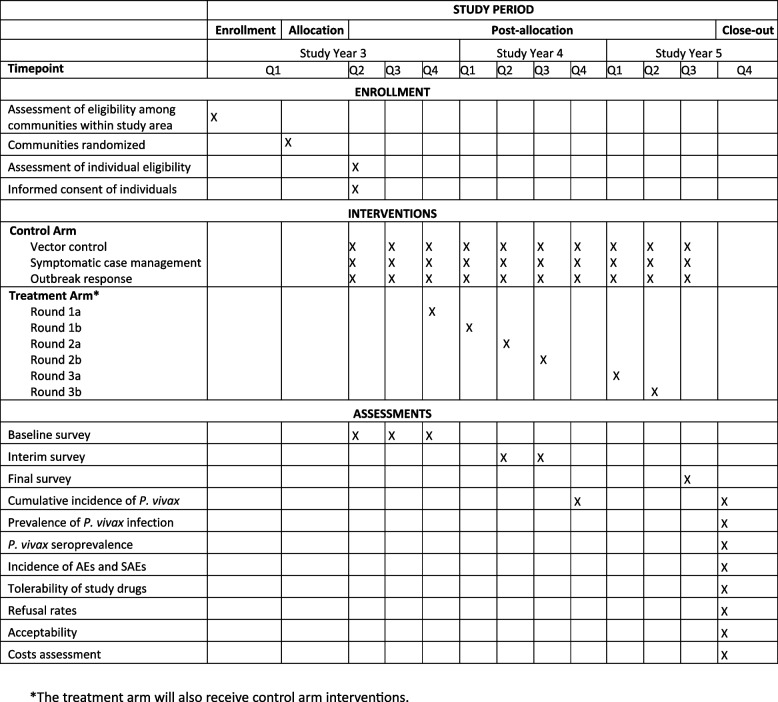
SPIRIT schematic to depict the schedule of enrollment, interventions, and assessments. Study years begin in June and end in May. The treatment arm will receive control arm interventions in addition to the treatment arm interventions

This is a 3-year open-label CRCT, including enrollment, allocation, post-allocation, and close-out phases (Fig. 2). The trial will be pragmatic and implemented through the existing health system. To demonstrate sustained impact in this tropical setting where strains of *P. vivax* relapse earlier than those once found in Central China [29], the fMDA intervention is relatively aggressive at two rounds per cycle, and the implementation period and follow-up will be longer than prior trials [1].

### Study setting and trial preparations

The trial will take place in Loreto Department, located in the Peruvian Amazon and where more than 90% of the country’s malaria cases occur [30]. Malaria transmission is perennial and historically peaks around April to June, though recently transmission has leveled out throughout the year. Based on national data from the first half of 2024, 82.5% of reported cases were *P. vivax* [30].

**Fig. 3:**
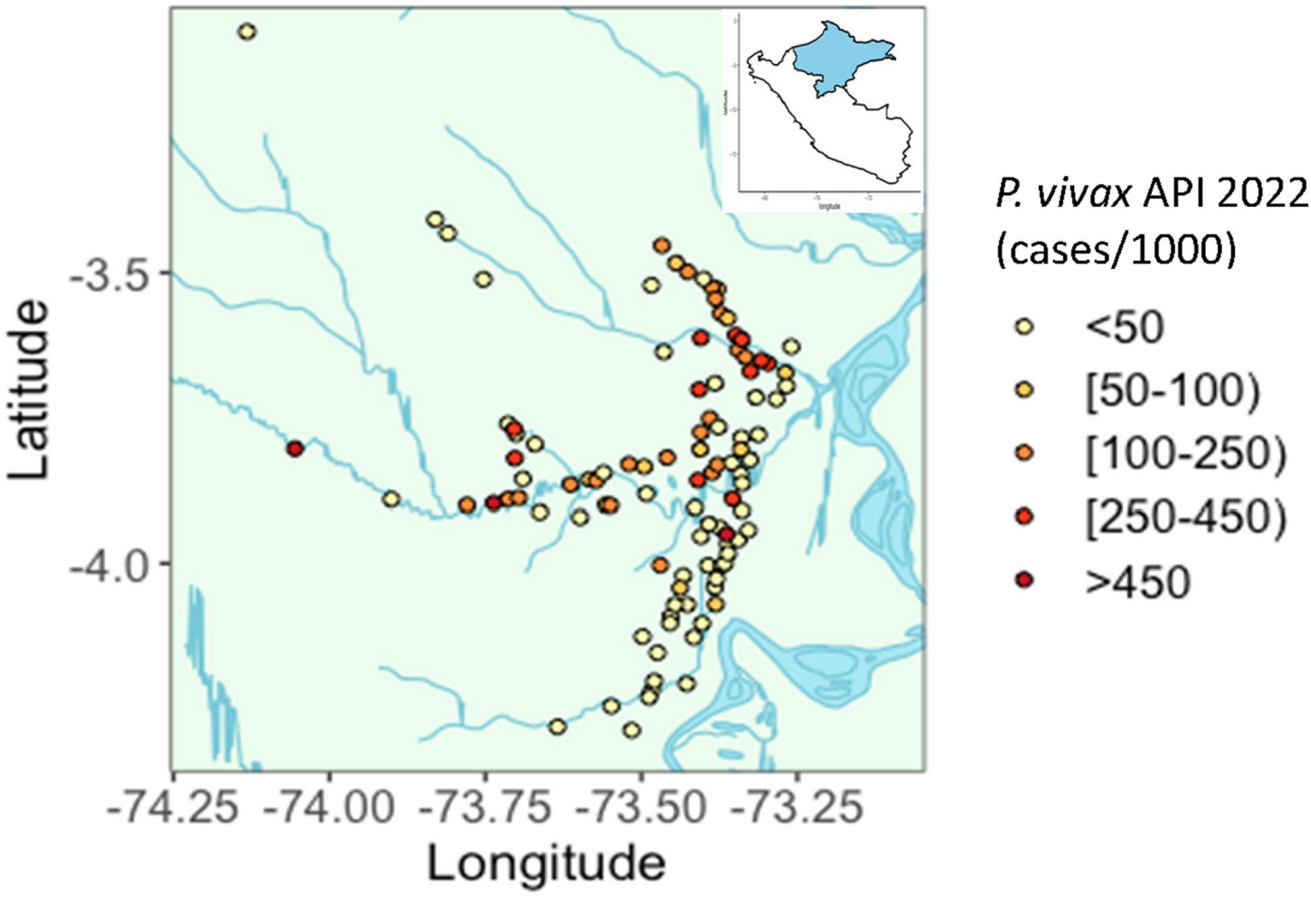
Map of communities within the study area by annual parasite incidence (API) in 2022. API calculated as annual number of malaria cases reported among residents of community per 1000 population. Population data is based on most recent population estimates from 2017 or 2019, depending on the community

Communities or clusters will be eligible if they are located within 8 h of riverine or road transport of Iquitos. Villages with high (API > 250/1000) or sporadic transmission (< 2 cases) in the year prior to the trial, or extreme population size (> 650) will be excluded. A map of the study area and distributions of recent *P. vivax* annual parasite incidences (API) among communities in the region are shown in Fig. 3.

Prior to the trial commencing, activities will be conducted to facilitate study implementation and gather baseline epidemiological data. A census and geographic reconnaissance survey was conducted in August 2023 to enumerate the study area and geolocate households. Malaria cases from the 2 years prior to the trial launch will be collated from paper-based malaria registers and NOTIWEB, the electronic malaria case reporting system. Based on these and past census data, cases in individuals residing in the study area will be geolocated to their household.

Prior to the trial launch, the study team will engage in community sensitization, which will consist of meeting with Gerencia Regional de Salud (Regional Management of Health) leadership and engaging with local community leaders, health workers, and villagers. Prior to trial launch, health facilities in both arms will receive refresher trainings regarding case management and case reporting to bolster the strength of the existing health surveillance program and ensure high surveillance data quality across both arms.

### Randomization and masking

The unit of randomization will be a community. A restricted randomization will be conducted taking into consideration *P. vivax* incidence in the prior year, distance to the nearest health post, distance to Iquitos, and population size. The Data Manager will remain unblinded to study arm assignment; however, the trial statistician and data analyst will be blinded to the study arm assignment.

### Procedures

#### Enrollment and a baseline assessment

Individual written informed consent will be sought during the baseline survey or during subsequent surveys for new participants. Verbal consent will be requested separately for each subsequent study procedure, including completion of questionnaires, blood testing, and drug administration. Consent for minors less than 18 years of age will be sought from a parent or guardian, and minor assent will also be sought from participants 8–17 years old. Informed consent will be sought in Spanish.

Enrollment and a baseline assessment in consenting individuals will be sought prior to any intervention implementation activities. At the baseline assessment, demographic, epidemiological, and clinical data will be collected. A finger prick will be conducted for rapid G6PD testing using the STANDARD G6PD test (SD Biosensor, Inc., Suwon, South Korea) per manufacturer’s instructions. G6PD status will be categorized as deficient ($\le$ 4.0 IU/g Hb), intermediate (4.1–6.0 IU/g Hb), or normal (≥ 6.1 IU/g Hb), which reflect ranges of enzymatic activity below or near normal activity of 9.0 IU/g Hb [31], respectively. As the STANDARD G6PD test includes a hemoglobin (Hb) measurement, this result will also be recorded. Blood from the same finger prick will also be used to generate a dried blood spot (DBS) for malaria molecular testing and G6PD sequencing to identify variants among G6PD deficient and intermediate individuals and 5% of the G6PD normal individuals. For individuals with fever in the past 48 h, microscopy will be performed by health promoters at health establishments, and treatment will be provided per national policy. Current first-line treatment is artesunate-mefloquine for *P. falciparum* and chloroquine (CQ, 10 mg/kg on days 1 and 2, and 5 mg/kg on day 3) plus PQ (0.5 mg/kg/day × 7 days) for *P. vivax* [32].

#### Standard malaria interventions in the control and intervention arms

As part of the country’s national malaria elimination program, Plan Malaria Cero (Plan Malaria Zero) [26], standard malaria interventions will be provided in both study arms. Led by health promoters, these interventions include passive and active case detection at community members’ homes or at health establishments, microscopy at health establishments, and treatment per national policy guidelines. Tafenoquine (300 mg × 1) is registered in Peru as a radical cure for individuals that are G6PD normal and $\ge$ 16 years of age, but it is currently only available in pilot health facilities outside of the study area. Passive case detection detects symptomatic cases at health facilities. Active case detection up to 6–8 times per year involves village-wide searching for fever, followed by treatment of individuals testing positive by microscopy or rapid test. Vector control interventions include provision of Duranet insecticide-treated bed nets (1.5–1.7 people/net) impregnated with alpha-cypermethrin provided every 3 years on average and spraying with pirimiphos-methyl insecticide approximately 2 times per year (78.2% coverage of households in the Maynas Province of the Loreto Department).

#### fMDA in the intervention arm

**Fig. 4:**
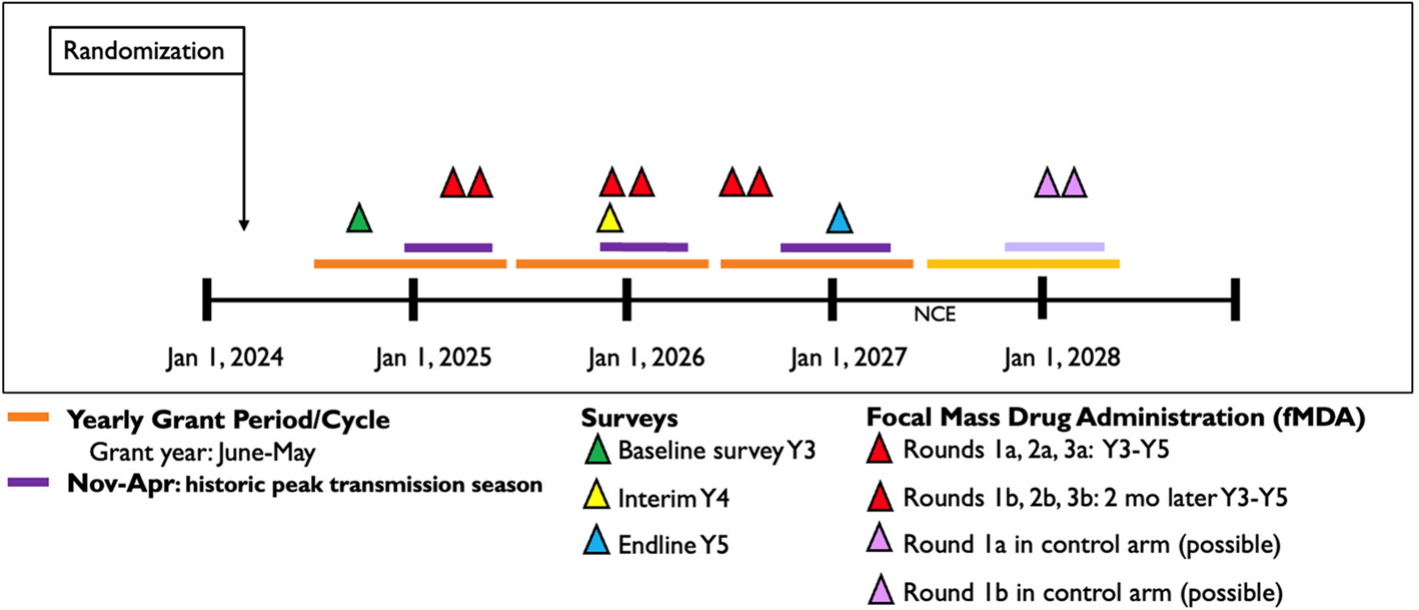
Schematic of the overall study timeline and major activities including surveys and fMDA. Each activity is demarcated with a colored triangle. The grant period includes annual cycles from June to May (orange bars), and the historic peak transmission season includes the period between November and April (purple bars). Abbreviations: NCE = no cost extension

In the intervention arm, fMDA will be administered over 3 consecutive years. In each cycle, the intervention arm will receive two rounds of fMDA, with each round separated by 2 months and each cycle separated by 9–11 months (Fig. 4). fMDA will be administered by directly observed therapy (DOT) and will target high-risk individuals meeting eligibility criteria detailed in Table 1. These eligibility criteria include being a malaria index case or living in a household within a 200-meter radius of a *P. vivax* index case. The 200-meter radius was selected based on evidence from other cohort studies in the Peruvian Amazon indicating clustering of asymptomatic, submicroscopic infections among neighboring households, generally within 200 meters [33–35]. Index cases will include cases reported in the 2 years prior to the first round of each fMDA cycle detected through passive or active case detection. Individuals with high-risk status will be screened for other inclusion and exclusion criteria for fMDA eligibility.

**Table 1:** Eligibility criteria for study activities

	Inclusion criteria	Exclusion criteria
Clusters	• Within 8 h transport of Iquitos • Incidence < 250/1000 and > 2 cases in the year prior to trial • Population size (< 650)	• Outside of study area including villages on the banks of the Momon, Nanay, Itaya, and Pintuyacu rivers and along the main road in the region, Carretera Iquitos-Nauta
Study enrollment	• Slept in household within study community at least one night in the past 4 weeks •$\geq$ 6 months old • Signed informed consent	
fMDA		
Chloroquine	• Resides in neighboring household but within 200 m of Pv index case in the past 2 years • Age ≥ 6 months old • Present for intervention • Adult ≥ 18 years old that provides informed consent • A child ≥ 8 years and < 18 years old that provides informed assent and has informed consent from their parents • A child ≥ 6 months old and < 8 years old that has informed consent from their parents	• History of retinal or visual field changes • Known hypersensitivity or adverse reaction to CQ • Currently taking CQ or have taken CQ in the past 4 weeks • Ineligible for TQ or PQ (see criteria below) • Hemoglobin < 9 g/dL
Tafenoquine	• Eligible to receive CQ • Age ≥ 16 years old • Adult ≥ 18 years old that provides informed consent • A child ≥ 16 years and < 18 years old that provides informed assent and has informed consent from their parents	• G6PD deficiency or intermediate status (defined as activity $\le$ 6.0 UI/gHb per SD biosensor) • G6PD status unknown or refusal of G6PD status test • Acute or severe malaria • Pregnancy (known or identified by pregnancy test) • Refusal of pregnancy test if new amenorrhea in the past 4 weeks • Woman breastfeeding a child that is G6PD deficient or with unknown G6PD status • Known hypersensitivity or adverse reaction to TQ or PQ • Have taken mefloquine (i.e., artesunate-mefloquine), TQ or PQ, or other antimalarial in the past 4 weeks • Hemoglobin < 9 g/dL
Primaquine	• Eligible to receive CQ and ineligible to receive TQ • Age ≥ 6 months old • Adult ≥ 18 years old that provides informed consent • A child ≥ 8 years and < 18 years old that provides informed assent and has informed consent from their parents • A child ≥ 6 months old and < 8 years old that has informed consent from their parents	• G6PD deficiency (defined as activity ≤ 4.0 UI/gHb per SD biosensor) • G6PD status unknown or refusal of G6PD status test • Acute or severe malaria • Pregnancy (known or identified by pregnancy test) • Refusal of pregnancy test if new amenorrhea in the past 4 weeks • Breastfeeding child with documented or unknown G6PD deficiency status • Known hypersensitivity or adverse reaction to TQ or PQ • Have taken mefloquine (i.e. artesunate- mefloquine), TQ or PQ, or other antimalarial in the past four weeks • Hemoglobin < 9 g/dL

Each round of fMDA will include CQ plus either TQ, for non-pregnant individuals $\ge$ 16 years of age and G6PD normal, or PQ, for non-pregnant individuals 6 months to 15 years of age and G6PD normal or intermediate. The prevalence of G6PD deficiency in the study area is anticipated to be < 5% [36–40]. Preliminary data from the study area shows that 36.2% of the study population is > 6 months and < 16 years and will receive PQ. If pediatric TQ is approved for use in Peru during the study, its use will be incorporated into the study.

For the first annual rounds (referred to as 1a, 2a, and 3a for years 1, 2, and 3, respectively) the regimen will include 3 days of CQ (10 mg/kg on days 1 and 2, and 5 mg/kg on day 3) for treatment of *P. vivax* asexual blood stages, with TQ or PQ for *P. vivax* hypnozoite stages (TQ 300 mg × 1 or PQ 0.5 mg/kg/day × 7 days) (Table 2). PQ and TQ provide prophylactic and gametocytocidal effects against both *P. vivax* and *P. falciparum* [41–45]. However, compared to PQ which has a short half-life of roughly 4–9 h [46–48], TQ will provide an extended period of protection given its prolonged terminal elimination half-life (~ 15 days), the time it takes to reduce the plasma concentration of TQ by 50%. This will provide post-treatment prophylaxis for up to 77 days [49, 50].

**Table 2:** Drugs formulations and doses for all fMDA Rounds

Drug*	**Dosage form (mg)**	**Administration**	**Manufacturer**	**Dose**	
				**≥16 years**	**6 mos – 15 years**
**Chloroquine (CQ)**					
3-day CQ (Rounds 1a, 2a, 3a)	150 mg base	Take with food to minimize possible gastrointestinal irritation	Kronos Lab, Guayaquil, Ecuador	Dose ~10 mg/kg (D1, D2), ~5 mg/kg (D3)	Dose ~10 mg/kg (D1, D2), ~5 mg/kg (D3)
sdCQ (Rounds 1b, 2b, 3b)				Dose ~10 mg/kg once	Dose ~10 mg/kg once
**Tafenoquine (TQ)** (all Rounds)	150 mg	TQ should be taken with food to increase absorption	Piramal Pharma Ltd, Mumbai, India	300 mg once	n/a
**Primaquine (PQ) **(all Rounds)	15 mg	Take with food	Colompack S.A., Bogota, Colombia	Dose ~0.5 mg/kg daily x 7 days (If TQ is contraindicated)	Dose ~0.5 mg/kg daily x 7 days
*fMDA: focal mass drug administration; CQ: chloroquine; sdCQ: single dose chloroquine; TQ: tafenoquine; PQ: primaquine; D1: Day 1; D2: Day 2; D3: Day 3* *The commercial name of the product and the manufacturer may vary depending on if the product is bought or donated.					

Coverage and effectiveness of MDA can be compromised by drug eligibility criteria and potential challenges of acceptability, drug efficacy, adherence, imperfect surveillance to identify high-risk individuals, and human movement. Additional rounds of MDA may be needed, particularly in tropical settings where strains cause more frequent relapses in patients. Thus, the fMDA intervention will include second annual rounds (referred to as 1b, 2b, and 3b for years 1, 2, and 3, respectively). 

The regimen will include single-dose CQ (10 mg/kg × 1) with either PQ (0.5 mg/kg/day × 7 days) or TQ (300 mg × 1). CQ, including in a single dose, may potentiate the anti-relapse effect of PQ and TQ [7, 51, 52]. A single dose in the second annual rounds will also facilitate administration and acceptability. Administration of this second round at approximately 2 months after each first annual round will serve to prolong the anti-relapse, as well as providing prophylactic and transmission-blocking activity of fMDA. In multi-site, double-blind, double-dummy, randomized trials comparing TQ to placebo and to PQ [31, 36], efficacy to prevent recurrences was 72.8% (95% CI 65.6–78.8) and 67% (95% CI 61.0–72.3) for PQ and TQ, respectively, at 180 days.

#### Safety

Adverse events (AE) include any unfavorable or unintended sign, symptom, disease, syndrome, abnormal laboratory finding, or illness that emerges or worsens relative to each participant’s pre-treatment baseline, whether or not it is connected to the study intervention. Study clinicians will assess and grade all AEs based on the DAIDS severity grading scale [53]. AEs will be monitored passively, by participant calls to study staff or visits to a local health facility, and actively, through study staff who will actively inquire about AE at monthly visits.

Lack of drug tolerability will be assessed as missed doses due to vomiting or non-adherence due to adverse effects. An AE of special interest (AESI) in this study is 8-aminoquionline-associated hemolysis which is known to occur in individuals with G6PD deficiency and with malaria itself. Monitoring for this AESI and other adverse reactions will be conducted through passive and active surveillance being conducted in both study arms. Additionally, active pharmacovigilance will occur as study staff inquire about symptoms among during return visits to complete treatment. After receipt of fMDA Round 1a, Hb and urine testing will be conducted on the same day between days 3 and 7 post first treatment with either PQ or TQ [8]. Hb testing will be conducted using the Hemocue portable spectrophotometer per manufacturer’s instructions [8]. Participants and their caretakers will be made aware of possible AE and associated potential symptoms. They will be instructed to act without delay given the possible severity of some events.

For hemolytic events or other severe AEs, participants will be transported within 4 h to the hospital in Iquitos where blood transfusion therapy and other tertiary care is available. Any participant that has any severe adverse reaction associated with any study drug will be withdrawn from receiving further study drug. The participant will continue to be followed as per protocol except will not receive any additional doses of study medication.

#### Identification of index cases and measurement of incidence

Malaria case information is recorded in fever books at health posts, and these cases are reported to the regional level. Index cases from the 2 years prior to trial launch and prior to each cycle of study medication administration will be geo-located prior to that cycle. Index cases also include cases detected during regular interventions by the health system and asymptomatic cases identified during study surveys.

During the trial, any participant with a fever will be instructed to seek medical care. For any new or relapse malaria cases diagnosed, a finger prick will be obtained to collect a DBS before administering treatment. A study team member will also conduct a case investigation (within 48 h of diagnosis) to confirm clinical and demographic details. If a pre-treatment sample was not obtained, study staff will aim to collect a sample from the participant within 3 days. If the new index case is an enrolled participant in the intervention arm, they will be eligible for fMDA at the beginning of the next cycle.

The primary outcome measure of cumulative incidence is the number of incident symptomatic microscopy-confirmed *P. vivax* malaria cases in residents divided by the total population. Cumulative cases will be calculated during the period from trial launch through 1 year after the launch of round 3a of fMDA. The denominator will include all the participants in the study except for those who have withdrawn from the study.

#### Interim and endline cross-sectional surveys

An interim survey in a sample of the enrolled population will be performed prior to the second fMDA cycle where participants are surveyed house to house. From the list of households enrolled at the time of the survey, a random sample of households will be selected proportional to cluster size. The final study year will conclude with an endline survey in all clusters. The goal of the surveys will be to invite new or unenrolled community members to enroll in the study to include a larger proportion of the population in the study, update demographic and clinical data from enrolled participants, and collect DBS to assess secondary trial outcomes of infection prevalence and seroprevalence. Acceptability of fMDA will also be assessed in the intervention arm. Acceptability will be assessed both quantitatively and qualitatively, in terms of refusal rates and open-ended questions in the intermediate and endline surveys.

#### Costing and cost-effectiveness assessment

Cost data will be captured in a quantitative survey provided to all participants who were diagnosed with a confirmed case of malaria by a health establishment during the trial period. The survey will be administered 15–30 days following the diagnosis to allow potential costs associated with the infection to accrue. Cost data will include direct intervention costs for the provider (e.g., Ministry of Health capital expenditures, consumables, personnel, training, transport, and infrastructure); direct costs to participants (e.g., other medications not provided free-of-charge by the interventions, transport costs); and indirect costs from the participant perspective (e.g., lost wages due to malaria per patient and companions).

#### Non-trial care

Participants will be free to seek usual and as-needed medical care at their own discretion, with no effect on study eligibility or arm allocation. Participants undergoing blood testing or receiving medication(s) will receive anticipatory guidance on potential side effects. In the event of symptoms, participants will be instructed to notify the study staff. Study teams encountering individuals with severe or uncomplicated malaria, or other acute illness, will refer such participants to the nearest health facility.

#### Data management

Data will be captured using REDCap forms on electronic tablets and uploaded daily to a secure server. When internet and/or electricity is unreliable, paper forms will be used to collect information instead of tablets. Training on how to capture electronic health data and use the tablets will be conducted before each survey and round of fMDA. Study documents will be retained for 10 years following the end of the trial.

#### Laboratory methods

##### Malaria microscopy

Participants will be instructed to seek care at local health posts whenever they have a fever, and malaria testing will be conducted by microscopic blood film examination. Blood smears will be stained with 2% Giemsa for 30 min and read by experienced microscopists. Parasite densities will be calculated from the number of asexual parasites per 200 leukocytes (or per 500, if < 10 asexual parasites/200 leukocytes), assuming a leukocyte count of 8,000/µL. A blood smear will be considered negative if examination of 100 high power fields does not reveal asexual parasites. Thin smears will be used for parasite species identification.

##### Molecular methods

Molecular diagnosis by real-time PCR will be performed following the modified protocol reported by Mangold, Manson et al. [54]. Parasite density calculations will be based on standard curves generated using clinical samples with known parasite concentration. Additionally, more sensitive methods may be used including amplification of parasitic mitochondrial DNA, which may improve sensitivity more than ten-fold compared to 18 s rRNA assays [54, 55]. The amplification of mitochondrial genes could be combined with the use of additional PCR targets to discriminate between species.

Genotyping of both the human and parasite DNA will be performed on blood samples to document G6PD and CYP2D6 genotypes in Peru, to assess within-host parasite genetic diversity, classify infections as local or imported [56], and classify *P. vivax* infections as new, recrudescent, persistent, or relapse [57]. G6PD variants previously associated with G6PD deficiency in Latin America as well as any new variants will be genotyped using long-read sequencing (Oxford Nanopore) of PCR amplicons covering 11 Kb of the ~ 18Kb gene (manuscript in preparation). Similarly, CYP2D6, variants which may impact metabolism and thus efficacy of 8-aminoquinolines, will be genotyped in a subset of participants with recurrent episodes after fMDA administration. Highly discriminative genotyping will be conducted by Illumina-based targeted deep sequencing of short, variable regions with numerous alleles (microhaplotypes) for *P. falciparum* and *P. vivax* [58–60].

##### Serological methods

Using methods described previously [61, 62], serological tests will be performed using samples from cross-sectional surveys to measure antimalarial antibodies that reflect recent and more distant exposure. Malarial antigens to assess distant exposure may include: *Pv* merozoite surface protein (MSP)−1.19, *Pv* apical membrane antigen (AMA)−1, *Pf* glutamate-rich protein-fragment (GLURP)-R2, and *Pf* AMA-1. Based on preliminary data from our cohort studies, malarial antigens to assess recent exposure may include other objectives such as, *Pv* MSP-10, *Pv* MSP-8, Pv RBP2b [63–65], and *Pf* Early transcribed membrane protein (Etramp)−5. A Gaussian mixture model will be used to determine positivity. Reversible catalytic conversion models fitted by standard maximum likelihood will be used to generate a seroconversion rate (SCR) [66, 67]. A longitudinal analysis in the same individuals will also enable study of the dynamics in malaria exposure following interventions [68].

## Outcomes and measures

### Sample sizes and power calculations

The study is designed to have 80% power to detect at least 68% relative reduction in cumulative *P. vivax* incidence for fMDA versus control over 3 years, with 15 clusters per study arm (mean population per cluster = 251), based on anticipated baseline *P. vivax* API of 65 cases/1000 population for the control arm, a coefficient of variation (*k*) of 0.87 based on past data, and a 0.05 significance level using a two-sided test [69]. As Peru is aiming for malaria elimination, this anticipated effect size is considered necessary and consistent with available evidence [18–20, 70, 71].

### Statistical analysis

#### Primary analysis

An intention-to-treat (ITT) approach will used, in which all clusters with at least one index case during follow-up will be analyzed according to their randomized intervention assignment. For the primary outcome of cumulative incidence of all microscopy-confirmed cases reported in study area residents, negative binomial regression using generalized linear models with cluster-level case counts and cluster population size as an offset will also be used to estimate cumulative incidence ratios (CIR) between study arms [70, 71]. The primary analysis will adjust for covariates used in the restricted randomization (baseline incidence, population size, distance to Iquitos, and distance to a health post) [72]. We will also perform a secondary adjusted analysis that controls for covariates used in the restricted randomization as well as baseline covariates (e.g., baseline coverage of vector control interventions) that are correlated with the outcome (likelihood ratio test *p*-value < 0.2). The secondary adjusted analysis may yield higher precision than models only adjusted for restricted randomization covariates due to any chance imbalances in baseline covariates that are associated with the outcome [73]. Comparisons of incidence measures will be expressed at the cumulative incidence ratio (CIR) or the protective efficacy (PE = 1-CIR × 100%) with their corresponding 95% confidence intervals.

#### Secondary analyses

For secondary outcomes of incidence, the same approach as described above will be used to generate CIRs or adjusted CIRs (aCIRs). For the outcome of clinical malaria, Kaplan–Meier survival curves will be produced. If survival is proportional between study arms, a time to event analysis using Cox proportional regression analysis will be conducted to estimate hazards ratios (HR). For outcomes with continuous variables, a linear regression will be conducted. For prevalence outcomes, log-binomial models will be fit to estimate prevalence ratios (PR). A modified Poisson regression with robust standard errors may be used if log-binomial models do not converge. For longitudinally measured outcomes (e.g., parasite prevalence, serology measures, refusal rates), generalized mixed-effect models will be constructed where participant/cluster random effects are included to account for correlation among observations from the same subjects and to account for the clustered study design. Intra-cluster correlation coefficients will be reported from these models. Time-intervention interaction will be evaluated to assess difference in trend between arms. Key subgroup analyses such as age, sex, baseline transmission intensity, distance to Iquitos, distance to a health post, and population size will be performed. All estimators that result from these models will be reported with corresponding 95% confidence intervals and p-values.

Per-protocol analyses will be restricted to clusters in which at least 80% of interventions were delivered according to the study protocol. Analyses will use g-methods, which are generalized methods that separately adjust for baseline and post-treatment covariates associated with participants not adhering to treatment [74].

#### Spillover effects

An effective intervention must decrease or interrupt transmission among individuals receiving the intervention (a “direct effect”) as well as among non-recipients outside of intervention zones (a “spillover effect” or “community effect”) [75–77]. There is a biological basis for spillover effects of fMDA. Chemotherapy can block transmission by reducing gametocyte biomass, and subsequent movement of humans and mosquitos can result in spillover effects outside of intervention zones. Our primary analysis at the cluster level is a pooled effect estimate across intervention recipients and non-recipients. Direct and spillover effects of fMDA will be estimated by comparing malaria incidence and prevalence in non-recipients in proximity to fMDA zones by arm. Direct effects will be defined as the cumulative incidence ratio (or prevalence ratio) among treated individuals in focal treatment zones within 200 meters of index case in the intervention arm vs. the control arm. Spillover effects will be defined as the cumulative incidence ratio among treated individuals outside focal treatment zones around index cases in the intervention arm vs. the control arm. To test for possible contamination of the control arm, using data from the endline prevalence survey, we will assess whether malaria prevalence in the control arm differs between households closer to or further from treatment clusters. We will calculate the distance from each household sampled in the endline survey to the nearest treatment cluster boundary. Then we will perform a clustered permutation test of the null hypothesis that malaria incidence and prevalence do not differ at the 20th and 80th percentiles of the distance to the nearest treatment cluster boundary, controlling for covariates used in the constrained randomization [78].

#### Safety and tolerability analysis

Serious adverse events refer to any expected or unexpected event, related or unrelated to the study medication, that results in death, a life-threatening event, hospitalization, prolongation of hospitalization, disability or incapacity, or a congenital anomaly. The incidence of serious adverse events (SAE) from fMDA, defined as SAEs divided by person-time at-risk, will be measured. Sub-analyses will be conducted by drug type. The incidence of SAE or severe malaria in fMDA will also be compared to the incidence of severe malaria in the control arm [79–82]^.^ AEs and adherence will also be assessed descriptively.

Lack of drug tolerability will be assessed as missed doses of study drugs due to vomiting or non-adherence due to adverse effects. Adverse event monitoring will be conducted both actively and passively.

#### Cost-effectiveness

A cost-effectiveness analysis will be conducted from both provider and patient perspectives. Health outcomes for participants allocated to fMDA and control interventions will be estimated using probabilistic decision tree models, and then compared to determine the incremental effects of fMDA in terms of *P. vivax* incident and prevalent infection averted and disability-adjusted life years (DALY) averted. The results of the trial will be the main source of data on probabilities and complemented with data from the malaria surveillance systems and the scientific literature. DALYs will be estimated using disability weights and life expectancies at death from the WHO life tables. Cost data will include direct and indirect costs of interventions. Costs will be adjusted for inflation and to reflect local government salaries. Deterministic and probabilistic sensitivity analyses will be conducted to understand the effect of parameter uncertainty (effect probabilities and costs) on the incremental cost-effectiveness ratio (ICER) of fMDA, expressed as the cost per *P. vivax* incident infection averted and cost per DALY averted. The analysis will be conducted at the end of the trial after all rounds of fMDA have been completed.

### Ethics

The study will be conducted in accordance with accepted principles on Ethics in Human Experimentation and ICH/GCP. Participants will be compensated a total of 30 soles (~ USD 8.20) per family per visit to compensate them for their time they contribute to the clinical trial away from their jobs. This money will be paid in cash and will be recorded in a document signed by the head of each family acknowledging their compensation. In order to protect the privacy of patients, only authorized study personnel will have access to paper records, and those records will be kept in a locked file at the site facility, Asociación Civil Selva Amazónica. Later they will be transported to the Malaria and Leishmania Unit at the IMTAvH—UPCH in Lima in compliance with established requirements (Reglamento de Ensayos Clínicos). No names will appear on any forms or publications. No information will be shared with anyone else outside of the study without participants’ permission.

### Monitoring and auditing

A fully independent data safety monitoring board (DSMB) will oversee the progress and safety of the study. The board includes a chair with expertise in cluster randomized controlled clinical trials, as Scientist with expertise in malaria, a biostatistician with expertise in clinical trials, and a local Scientist with experience in data and safety monitoring of trials. The DSMB will be included in discussions prior to the start of the trial to review the analysis plan and will meet approximately annually throughout the trial to advise on study progress. The DSMB may decide to withdraw clusters or participants, including cessation of activities for those clusters or participants. Should early evidence of intervention safety problems arise, the DSMB will advise on stopping the study. The study will be halted if > 11/1000 individuals have incidence of any SAE, including by not limited to AHA, related to taking an 8-aminoquinoline. This stopping rule is based on WHO safety threshold guidelines [1].

Both an internal monitor and an external monitor will oversee the clinical trial. The external monitoring agency will conduct regular site visits including to the laboratory. The internal monitor will help develop a detailed monitoring plan including when/how to review patient charts, who will conduct monitoring visits, and who will address findings from monitoring.

The FLAME study team holds regular meetings to communicate about study progress and procedures. The study data management team meets monthly to monitor data quality. The entire study team holds meetings approximately quarterly.

## Discussion

### Potential challenges

A key challenge that may be encountered is ensuring high coverage of the intervention. Community members may be gone for days at a time working in the field, sleep in multiple homes, or live most of the time in the city of Iquitos. To maximize coverage of fMDA, medications will be delivered door-to-door by DOT at times convenient to the participant. Villagers that miss the first round of fMDA in a cycle or become eligible between the first round and second round will be re-considered for fMDA eligibility prior to the subsequent cycle.

Capturing all incident cases of malaria is also challenging. Participants may not seek care when they feel ill or may have an asymptomatic malaria infection. In order to capture as many cases as possible, households will be visited on a monthly basis by the field team to inquire about symptom onset or case diagnosis. Case report forms used to collect these data are include clear, time-anchored prompts that help participants accurately recall events to reduce recall bias. DBS samples will be collected from all study participants at two timepoints (baseline and endline surveys) which may aid in the detection of subclinical infections. Participants will be encouraged to seek care if they feel ill during the study.

The study communities in which we will intervene are dynamic and mobile in nature. The platform QField will be used to update maps of the study communities each visit and participants will be asked during monthly household surveillance if any individuals in their home are new or have left. In this way, population estimates will be maintained as accurately as possible.

## Trial status

Recruitment of study participants began on 14 October 2024 utilizing Spanish protocol version 5.6 from 9 September 2024. Initial enrollment efforts were completed in March 2025; however, participants may be enrolled throughout the trial until the completion of the trial in May 2026.

## Supplementary Information

Supplementary Material 1.

Supplementary Material 2.

## Data Availability

University of California San Francisco and Universidad Peruana Cayetano Heredia have shared access to the final trial dataset.

## Abbreviations

CQ: Chloroquine
CRCT: Cluster randomized control trial
DALY: Disability-adjusted life year
DOT: Directly observed therapy
DSMB: Data safety monitoring board
FLAME: Focal mass drug administration for vivax malaria elimination
fMDA: Focal mass drug administration
G6PD: Glucose-6-phosphate dehydrogenase
Hb: Hemoglobin
MDA: Mass drug administration
NCE: No-cost extension
PQ: Primaquine
Pv: Plasmodium vivax
TQ: Tafenoquine
WHO: World Health Organization
