# Supplementary material for: FocaL mass drug administration for Plasmodium vivax malaria elimination (FLAME): study protocol for an open-label cluster randomized controlled trial in Peru
Source: Trials. 2025 Oct 14;26:408. doi: 10.1186/s13063-025-09112-1 (PMC12522803; doi:10.1186/s13063-025-09112-1)
Supplement: Supplementary file 1 — Supplementary Material 1. [file 13063_2025_9112_MOESM1_ESM.docx]

**Supplemental Table 1.** Study outcomes

| **Aim** | **Outcomes** | **Definition** |
| --- | --- | --- |
| General | Cumulative incidence of *P. vivax** (Primary outcome) †‡ | Number of microscopy-confirmed, *P. vivax* malaria cases in residents** reported from health facilities per population over the follow-up study period |
| General | Prevalence of *P. vivax* infection*†‡ | Proportion of individuals with PCR-confirmed infection in an endline survey |
| General | *P. vivax* seroprevalence and seroconversion rates*†‡ | Proportion seropositive; Rate at which seronegative individuals became seropositive estimated from age-specific seroprevalence from endline survey, adjusted by modelling longitudinal individual serological status and/or antibody titers |
| General | Genetic diversity of *P. vivax* *†‡ | Diversity and relatedness of locally acquired infections |
| General | Prevalence of seropositivity†‡ | Proportion of individuals that are seropositive in the final endline cross-sectional survey |
| General | Prevalence of seroconversion†‡ | Proportion of individuals that seroconverted from baseline in the final endline cross-sectional survey |
| Specific 1 | Incidence of grade 3-4 AE or SAE | Based on a standardized AE grading scale^91^ (in fMDA arm only) |
| Specific 1 | Incidence of any AEs | As above |
| Specific 1 | Incidence of SAE and severe in fMDA, and incidence of severe malaria in the control arm | As above, severe malaria definition based on WHO criteria^62^ (inclusion of severe malaria will include a comparison across both study arms)^87–90^ See Appendix B for severe malaria criteria. |
| Specific 1 | Tolerability of study drugs† | Vomiting following administration of study drugs and measures of non-adherence |
| Specific 2 | Refusal rates† | Number of refusals divided by number of individuals invited to participate |
| Specific 2 | Acceptability† | Perceptions of intervention and reasons for participation assessed during the follow-up surveys and endline survey. |
| Specific 2 | Costs per unit (per fMDA round, per individual receiving intervention, per capita)† | Total costs divided by number of MDA rounds, number of individuals receiving intervention, and population of study area |
| Specific 2 | Cost per *P. vivax* case averted, disability-adjusted life-year (DALY) averted, or economic dollar saved*† | Difference in cost between fMDA and control divided by the difference in the effect (including incidence, prevalence, DALYs or economic dollar due to malaria) |

* Additional analyses will be conducted for *P. falciparum* only, and *P. vivax and P. falciparum, and restricted to cases classified as local based on travel and genetic data*

† Additional analyses will be conducted by year

‡ Additional analyses will measure direct effects and indirect (or “spillover”) effects, ie. outcomes in people that live near to but did not directly receive fMDA.

**Usual resident, or the village is where they live and sleep most of the time (more than any other village).
